# Comparing the antidepressant-like effects of electroconvulsive seizures in adolescent and adult female rats: an intensity dose–response study

**DOI:** 10.1186/s13293-023-00552-5

**Published:** 2023-09-30

**Authors:** Sandra Ledesma-Corvi, M. Julia García-Fuster

**Affiliations:** 1 IUNICS, University of the Balearic Islands, Cra. de Valldemossa Km 7.5, 07122 Palma, Spain; 2 Health Research Institute of the Balearic Islands (IdISBa), Palma, Spain; 3 Department of Medicine, University of the Balearic Islands, Palma, Spain

**Keywords:** ECT, Sex, Antidepressant, Neurogenesis, Hippocampus, Age

## Abstract

**Background:**

The induction of electroconvulsive seizures (ECS) in rodents induces sex- and age-specific disparities in antidepressant-like responses, with females and young age being the most unresponsive ones. Since the electrical charge needed to induce an effective convulsion is also altered by these variables, our aim was to compare different dose-intensities of ECS exclusively in female rats, since there is a lack of preclinical data characterizing this particular sex, while also evaluating efficacy during distinctive age periods of treatment (adolescence vs. adulthood).

**Methods:**

Adolescent and adult female Sprague–Dawley rats were exposed to an intensity dose–response study (55, 75 or 95 mA; 0.6 s, 100 Hz, 1 session/day, 5 days). The particular characteristics of the induced convulsions (tonic, clonic, recovery times) were monitored during treatment. Antidepressant-like responses were evaluated under the stress of the forced-swim test 1-, 3-, and 7-days post-treatment (i.e., improved immobility time as an indicative of an antidepressant-like response), and brains were collected 24 h later (8 days post-treatment) to evaluate potential changes in hippocampal neurogenesis (Ki-67 and NeuroD) by immunohistochemistry.

**Results:**

The lowest intensities tested of ECS (55 and 75 mA) induced an antidepressant-like effect in adult female rats, but rendered insufficient in adolescence. The lack of efficacy observed in adolescent rats paralleled differences in the characteristics of the seizures induced by ECS as compared to adulthood. In line with prior results, different dose-intensities of ECS modulated hippocampal neurogenesis in a comparable fashion with age (i.e., increased survival of neural progenitors 8 days post-treatment).

**Conclusions:**

In conjunction, these results reinforce the importance of fine-tuning the parameters of ECS that might render efficacious while considering sex and age as essential variables for treatment response, and suggest that other molecular mechanisms, beside the partial role of hippocampal neurogenesis, might be participating in the antidepressant-like effects induced by ECS.

## Plain Language Summary

Although the induction of electroconvulsive seizures (ECS) is a safe therapeutical option for treatment-resistant depression, there are important differences in treatment response driven by biological sex and age that require further characterization for ensuring optimal outcomes. In fact, most of the preclinical literature is centered in adult male rodents, with almost no prior studies characterizing ECS' response in adolescent female rodents. In this context, the present study compared the antidepressant-like responses induced by different intensity doses of ECS (55, 75 or 95 mA; 0.6 s, 100 Hz, 1 session/day, 5 days), exclusively in female rats (adolescent and adult). The results showed that the lowest doses tested (55 or 75 mA) induced an antidepressant-like response in adult female rats, while no dose was capable of inducing efficacy in adolescent female rats. These results replicated prior data from our group showing the inefficacy of the 95-mA dose at both ages, while demonstrating that lowering the dose is sufficient to exert efficacy in female adult rats. Further studies should center in adjusting the parameters to elicit efficacy in females during adolescence.

## Highlights

- There are clear sex and age differences in the antidepressant-like effects of ECS.
- Low dose intensities of ECS exerted antidepressant-like effects in adult female rats.
- ECS, at the doses tested, did not induce behavioral changes in adolescent female rats.
- ECS increased neurogenesis independently of the dose and age of treatment.

## Background

Electroconvulsive therapy (ECT) is a well-established treatment option for adult patients with treatment-resistant depression (e.g., [1, 2]), which is defined as the failure to respond to at least two different antidepressant treatments within a certain time and that affects around 20–30% of patients with major depressive disorders (MDD) (e.g., [3, 4]). However, the use of ECT in child and adolescent populations is less common and remains frequently unavailable, even though up to 60% of these young patients do not respond satisfactorily to first-line treatments (e.g., [5]) and despite the fact that ECT is generally considered safe at early ages (as detailed in the book edited by [6]). In an attempt to provide novel treatment strategies for adolescents with MDD, a recent systematic review [7] concluded that ECT was safe and effective for the treatment of mood disorders in child and adolescent populations. In conjunction with other recent reports that also described and/or revised the outcome of ECT in adolescents with MDD (e.g., [8–12]), the general recommendation from all of them would be that ECT should be considered and more broadly use in severe and treatment-refractory cases for adolescence.

The efficacy, safety and applicability of current ECT practices are the result of a series of improvements in treatment delivery, which have focused on preserving and improving efficacy (e.g., by adjusting ECT electrical dose, stimulus parameters and/or electrode placement), while minimizing the potential cognitive side effects (e.g., [13, 14]). Moreover, these parameters have to be adjusted by age and sex, since previous studies reported variations in the electrical charge needed to induce an effective convulsion (e.g., [15–19]): for example, women seem to require less charge than men of the same age to induce an optimal seizure, and for both sexes the charge needs to be increased with age (e.g., [20]). Interestingly, these differences can be modeled in experimental rodents through the induction of electroconvulsive seizures (ECS). In fact, a recent study from our group demonstrated age- and sex-specific differences in the antidepressant-like potential of repeated ECS (95 mA for 0.6 s at a frequency of 100 Hz square wave pulses, pulse width 0.6 ms, 5 days, 1 shock/day), since it worked when administered during adolescence or adulthood in male rats (although with a shorter length in adolescence as compared to adulthood), while in females rendered deleterious during adolescence (naïve rats) and ineffective in adulthood (maternally deprived rats; see [21]). Against this background the present follow-up study aimed at evaluating alternative dosing parameters (dose intensity study: 55, 75 and 95 mA) that could potentially induce an effective-like response in female rats, while comparing their response by age (adolescence vs. adulthood). Moreover, the next step evaluated the regulation of cell markers involved in the early stages of hippocampal neurogenesis (i.e., cell proliferation and early neuronal survival) as a potential mechanism behind the effects induced by ECS in female rats (e.g., [21]).

## Methods

### Animals

**Fig. 1:**
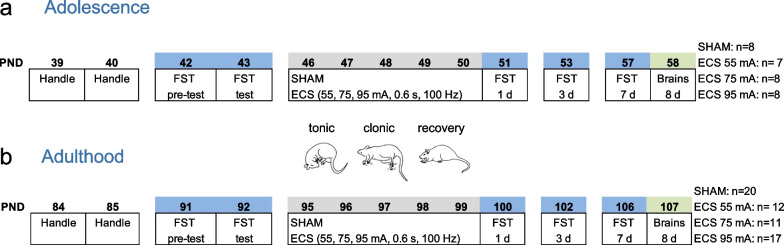
Experimental timeline. Effects induced by different intensity doses of ECS (55, 75 or 95 mA for 0.6 s, 100 Hz, 5 days, 1 dose/day vs. SHAM-treated rats) in **a** adolescent or** b** adult female rats. D, day of post-treatment; ECS, electroconvulsive seizures; FST, forced-swim test; PND, post-natal day

In this study we utilized exclusively female Sprague–Dawley rats (31 adolescents and 60 adults; Fig. 1) bred in the animal facility at the University of the Balearic Islands. Rats were housed in the vivarium (22 °C, 70% humidity, 12:12 h light/dark cycle, lights on at 8:00 h and off at 20:00 h) in standard cages with 2–4 animals with continuous access to a standard diet and tap water. All procedures were performed following the ARRIVE guidelines [22] and the EU Directive 2010/63/EU of the European Parliament and of the Council after approval by the Local Bioethical Committee (University of the Balearic Islands) and the regional Government (Conselleria Medi Ambient, Agricultura i Pesca, Direcció General Agricultura i Ramaderia, Govern de les Illes Balears). The specific stages of the estrous cycle were not monitored during the experimental procedures since the cyclicity of females was not part of our research question (see [23, 24]), but also because female rodents are not more variable than male rodents (e.g., [25]) and their spontaneous behavior might reflect individual variation rather than estrous state (see recent article by [26]).

### Electroconvulsive seizures (ECS)

Female rats from each age group (adolescence, postnatal days, PND 46–50, Fig. 1a; adulthood, PND 95–99, Fig. 1b) were exposed to a total of 5 shocks (1 per day during the light period: between 10:00 and 12:00 h) using a pulse generator (ECT Unit 7801; Ugo Basile, Italy) at different intensities (groups of 55, 75 or 95 mA) for 0.6 s at a frequency of 100 Hz square wave pulses and a pulse width of 0.6 ms, through earclip electrodes during independent experimental studies. While the intensity of 95 mA was selected from our own prior studies [21, 27], the lower intensities of 55 and 75 mA were based on other studies (e.g., [28–30]). All control rats were connected to the electrodes with no electrical current (SHAM groups; Fig. 1). The lengths (s) of the tonic and clonic seizure activities, as well as the recovery time were monitored on days 2, 3 and 4 of treatment by an experimenter blind to the treatment groups (see Fig. 1). Each time was calculated from the end of the prior phase, not as an overall time from ECS exposure. To estimate the mean time adolescent or adult female rats spent in each phase, we calculated an average value for days 2, 3 and 4 and treatment for each rat, which was utilized to calculate the mean overall value.

### Forced-swim test

Rats were screened in the forced-swim test to obtain their basal immobility rates (e.g., [21]). To do so, all rats were individual placed in water tanks (41 cm high × 32 cm diameter, 25 cm depth; temperature of 25 ± 1 °C) during 15 min (pre-test session; PND 42 or PND 91). The next day, rats were forced again to swim for a 5-min test session that was videotaped. Immobility rates were calculated for each rat (Behavioral Tracker software, CA, USA) and used to allocate rats in separate experimental groups that were counterbalanced by immobility (see Fig. 1). Since this test has been the goal standard screening tool in the industry for antidepressant-like responses (e.g., [31]), and is still widely used for screening novel potential antidepressants (e.g., [32]), later on, the behavioral response induced by ECS was evaluated 1-, 3- and 7-days post-treatment by re-exposing rats to 5 min sessions in the forced-swim test (as followed in our prior experimental procedures [21]). This repetitive screening testing provided reliable measurements of the behavioral response across time (see [21, 32–35]).

### Immunohistochemical analyses

Rats were killed by rapid decapitation 8 days post-treatment; note that while all adolescent rats were used for this analysis (Fig. 1), only a subgroup of adult rats were collected (SHAM, n = 11; ECS-55 mA, n = 12; ECS-75 mA, n = 11; ECS-95 mA, n = 8). The left half-brain was rapidly frozen in − 30 °C isopentane and then stored at − 80 °C until the whole extent of the hippocampus (-1.72 to -6.80 mm from Bregma) was cryostat-cut in 30 µm serial sections. For each rat, we collected 3 series of 8 slides, with each slide containing 8 tissue-sections, from the most anterior, middle and most posterior part of the hippocampus respectively, and as routinely performed for over 10 years (e.g., [21, 36, 37]). Subsequently, we utilized 3 slides (1 from each series, 24 tissue-sections in total) per marker (Ki-67 for cell proliferation and NeuroD for neuronal progenitors) in which to perform immunohistochemical analysis in the whole extent of the hippocampus as previously described [21, 36, 37]. Briefly, tissue was post-fixed in 4% paraformaldehyde to be later exposed to several steps such as epitope retrieval (only for Ki-67) and/or incubation with a peroxidase solution, and blocking in BSA. Later on, tissue was incubated overnight with the appropriate primary antibody (i.e., polyclonal rabbit anti-Ki-67, 1:20,000, kindly provided by Dr. Huda Akil and Dr. Stanley J. Watson, University of Michigan, MI, USA; goat anti-NeuroD1, 1:10,000; R&D Systems, Inc. a Bio-Techne Brand, MN, USA), followed by, the next day, a 1-h incubation with 1:1000 of biotinylated anti-rabbit or anti-goat secondary antibody (Vector Laboratories, CA, USA). To visualize Ki-67 or NeuroD + cells, we utilized an Avidin/Biotin complex (Vectastain Elite ABC kit; Vector Laboratories) and the chromogen 3,3′-diaminobenzidine (DAB) (with nickel chloride for NeuroD); when detecting Ki-67 + cells, tissue was counterstained with cresyl violet. Finally, all tissue was dehydrated in graded alcohols, immersed in xylene and cover-slipped with Permount^®^.

To quantify the number of immunostained positive cells we first coded the slides so the experimenter was blind to the experimental groups. Then, Ki-67 or NeuroD + cells were counted with a 63× objective lens and 10× ocular lens (amplification of 630×) under a Leica DMR light microscope, and following a modified unbiased protocol [38, 39] that counts every 8th section in the entire hippocampal dentate gyrus (for further details on the quantification or the method followed please check our prior study led by [21]). Finally, the number of Ki-67 or NeuroD + cells obtained for each rat was multiplied by the sampling factor 8 to provide a final estimate of the total + cells per marker and rat (see [36, 37]; also see [21] for further details on the quantification method).

### Statistical analysis

Data was analyzed and graphs were plotted with GraphPad Prism, Version 9.5 (GraphPad Software, CA, USA). Results are displayed as bar graphs incorporating mean values ± standard errors of the mean (SEM), and symbols for individual values for each rat (e.g., see guidelines for reporting data and statistical results in experimental pharmacology; [40, 41]). When comparing the properties of the convulsions elicited by the different intensities applied by ECS, we used two-way repeated measures (RM) ANOVAs with ECS intensity and Day of treatment as independent variables, or two-way ANOVAs with ECS intensity and Age as independent variables followed by Sidak's *post-hoc* test when appropriate. One-way ANOVAs were used to ensure that there were no basal changes in the time spent in the different behaviors (immobility, climbing, swimming) in the forced-swim test prior to assigning rats to the different experimental groups and starting ECS treatment. To evaluate the effect induced by different intensities of ECS treatment across days, data was analyzed by two-way RM ANOVAs, with Treatment (SHAM, ECS-55 mA, ECS-75 mA, ECS-95 mA) and Day post-treatment used as independent variables. Finally, changes in Ki-67 and NeuroD + cells were evaluated through one-way ANOVAs. Note that we did not include age (adolescence vs. adulthood) as an independent variable because experiments at each age period were performed at different time points in time, and therefore brain samples were not collected in parallel. This might have caused, as we previously described in our hands, differences in the basal rate of cell genesis among waves of experimental groups, driven by particular environmental conditions (see [42]). Dunnett’s multiple comparisons tests were used to compare each ECS intensity with the corresponding SHAM group. The level of significance was set at *p* ≤ 0.05.

## Results

### Characteristics of the seizures induced by different intensities of ECS treatment in adolescent and adult female rats

**Fig. 2:**
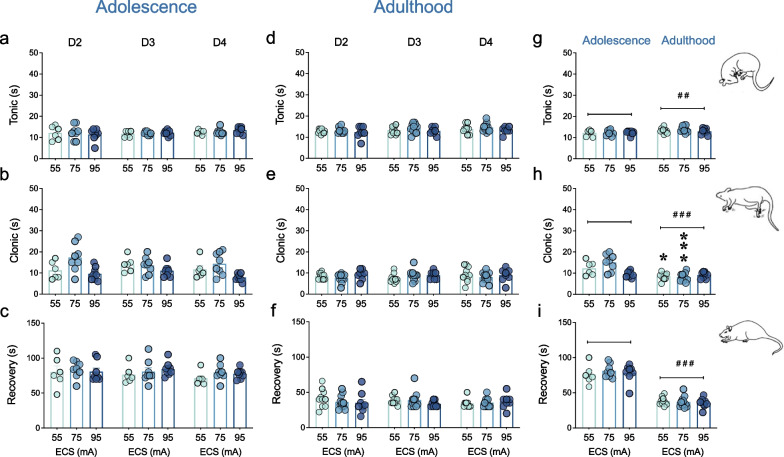
Characteristics of the seizures induced by different intensities of ECS treatment in adolescent and adult female rats. Time spent in tonic (s) and clonic (s) phases, or in recovery (s) during ECS exposure in (**a**–**c**) adolescence and (**d**–**f**) adulthood across days 2 and 4 of treatment, or (**g**–**i**) when comparing the average of all days for each phase in adolescence vs. adulthood. Data represents mean ± SEM of the time (s) spent in each phase. Individual values are shown for each rat (symbols). Two-way RM ANOVAs did not detect any significant changes in adolescence or adulthood. Two-way ANOVAs (independent variables: ECS intensity, Age) followed by Sidak's multiple comparisons tests: *p < 0.05 and ***p < 0.001 vs. same intensity-dose adolescent rats. Significant effects of Age: ^##^*p* < 0.01 and ^###^*p* < 0.001 when comparing adulthood vs. adolescence

All of the different intensities of ECS tested resulted in a period of tonic–clonic seizure activity, both in adolescent and adult female rats. However, when assessing the effects of the different intensities utilized for ECS to elicit the convulsions during adolescence, the results showed no significant ECS intensity x Day of treatment interactions for the time rats spent in tonic (F_4,38_ = 0.56, *p* = 0.696, Fig. 2a) or clonic (F_4,38_ = 1.86, *p* = 0.137, Fig. 2b) activities, nor in recovery (F_4,38_ = 0.40, *p* = 0.805, Fig. 2c). In particular, the average time an adolescent female rat exposed to ECS (independently of the ECS intensity or day, and by pooling all rats together) spent in tonic phase was of 12.3 ± 0.3 s (Fig. 2a), followed by 12.3 ± 0.3 s in clonic seizure activity (Fig. 2b), and 79.6 ± 2.1 s for recovery (Fig. 2c). Similarly, for adult female rats there were no changes in the properties of the convulsions induced by different ECS intensities across treatment days (tonic: F_4,52_ = 0.11, *p* = 0.979, Fig. 2d; clonic: F_4,52_ = 1.40, *p* = 0.247, Fig. 2e; recovery: F_4,52_ = 1.13, *p* = 0.354, Fig. 2f). For this age range, however, the average time an adult female rat exposed to ECS spent in tonic phase was of 13.5 ± 0.3 s (Fig. 2d), followed by 8.5 ± 0.3 s in clonic seizure activity (Fig. 2e), and 36.9 ± 1.3 s for recovery (Fig. 2f).

If comparing the time adolescent or adult female rats spent in each phase, two-way ANOVAs, with ECS intensity and Age as independent variables, found significant effects of Age for all variables (tonic: F_1,45_ = 8.81, ^##^*p* = 0.005, Fig. 2g; clonic: F_1,45_ = 29.42, ^###^*p* < 0.001, Fig. 2h; recovery: F_1,45_ = 239.5, ^###^*p* < 0.001, Fig. 2i), but only an effect of ECS intensity (F_2,45_ = 4.14, *p* = 0.022), as well as a significant interaction ECS intensity x Age (F_2,45_ = 7.91, *p* = 0.001) for the clonic phase (Fig. 2h). Particularly, Sidak's multiple comparisons test found that adult female rats spent significant lower times in the clonic phase (55 mA: − 3.8 ± 1.2 s, *p = 0.011; 75 mA: − 6.9 ± 1.1 s, ***p < 0.001) as compared to the adolescent ones (Fig. 2h).

### Behavioral responses scored under the stress of the forced-swim test following different intensities of ECS treatment in adolescent and adult female rats

**Fig. 3:**
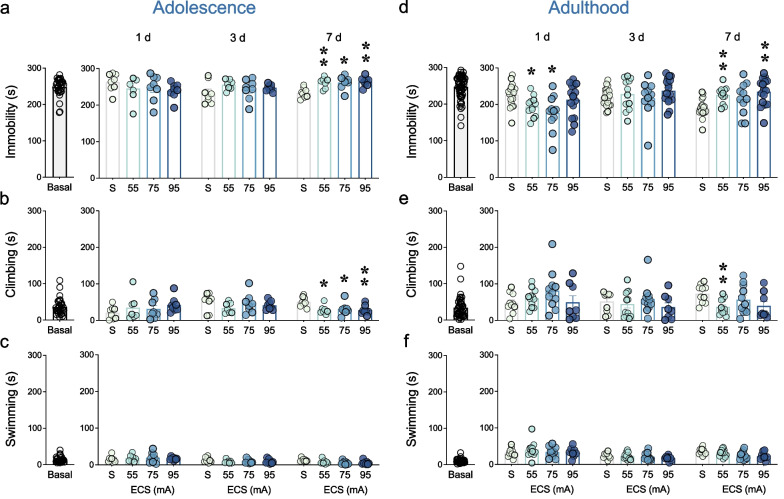
Behaviors scored under the stress of the forced-swim test following different intensities of ECS treatment in adolescent and adult female rats. Time spent in immobile (s), climbing (s) or swimming (s) basally (prior to treatment) or after ECS treatment (1-, 3- and 7-days post-treatment) in (**a**–**c**) adolescence and (**d**–**f**) adulthood in the forced-swim test. Data represents mean ± SEM of the time (s) spent in each behavior. Individual values are shown for each rat (symbols). Two-way RM ANOVAs followed by Dunnett's multiple comparisons tests: *p < 0.05 and **p < 0.01 vs. SHAM-treated rats (S) at the indicated post-treatment day

The average times spent in each one of the behaviors scored in the forced-swim test were similar for adolescent (immobility: 247.9 ± 4.4 s; climbing: 36.3 ± 4.0 s; swimming: 14.2 ± 1.4 s) and adult (immobility: 247.4 ± 4.1 s; climbing: 34.4 ± 4.4 s; swimming: 10.2 ± 0.8 s) rats (see Fig. 3). Rats were allocated in groups by cage and balanced by immobility to generate the treatment groups as detailed in Fig. 1. One-way ANOVAs did not detect any significant changes among the rats assigned to each experimental group for any of the behaviors evaluated (immobility, climbing or swimming; data not shown in graphs).

Adolescent ECS exposure modified the time female rats spent immobile in the forced-swim test (Treatment x Day interaction: F_6,54_ = 3.78, *p* = 0.003). In particular, *post-hoc* analysis revealed that ECS increased immobility (i.e., indicatives of a prodepressant-like effect) as measured 7 days post-treatment (55 mA: + 27.5 ± 7.4 s, **p = 0.007; 75 mA: + 27.8 ± 8.0 s, *p = 0.011; 95 mA: + 30.6 ± 6.7 s, **p = 0.001) vs. SHAM-treated adolescent female rats (Fig. 3a). As expected, ECS also altered climbing (Treatment x Day interaction: F_6,54_ = 3.92, *p* = 0.003), showing decreased rates 7 days post-treatment (55 mA: − 22.1 ± 6.7 s, *p = 0.015; 75 mA: − 20.3 ± 7.6 s, *p = 0.048; 95 mA: − 22.9 ± 6.5 s, **p = 0.009) vs. SHAM-treated adolescent female rats (Fig. 3b). Lastly, no Treatment x Day interaction was observed for swimming behavior (F_6,54_ = 2.11, *p* = 0.067; Fig. 3c).

Adult ECS exposure altered the time female rats spent immobile in the forced-swim test (Treatment x Day interaction: F_6,108_ = 9.08, *p* < 0.001), suggesting an antidepressant-like effect detected 1 day after treatment, both for the 55 mA (− 31.7 ± 10.7 s, *p = 0.018) and 75 mA (− 49.6 ± 16.2 s, *p = 0.022) intensities and as compared to SHAM-treated rats (Fig. 3d). Besides, rats exposed to ECS showed increased immobility (i.e., as also observed in adolescent female rats) 7 days post-treatment (55 mA: + 35.8 ± 8.7 s, **p = 0.001; 95 mA: + 44.8 ± 10.9 s, **p = 0.001) vs. SHAM-treated adult female rats (Fig. 3d). As expected, ECS modulated the time rats spent climbing (Treatment x Day interaction: F_6,74_ = 3.63, *p* = 0.003), and contrarily to what was observed for immobility, the results showed overall increased climbing rates 1-day post-treatment and decreased climbing 7 days post-treatment (55 mA: − 36.2 ± 9.9 s, **p = 0.005) vs. SHAM-treated adult female rats (Fig. 3e). Finally, no Treatment x Day interaction was observed for swimming behavior (F_6,74_ = 0.81, *p* = 0.567; Fig. 3f).

### Neurogenic-like effects induced by different intensities of ECS treatment in adolescent and adult female rats

**Fig. 4:**
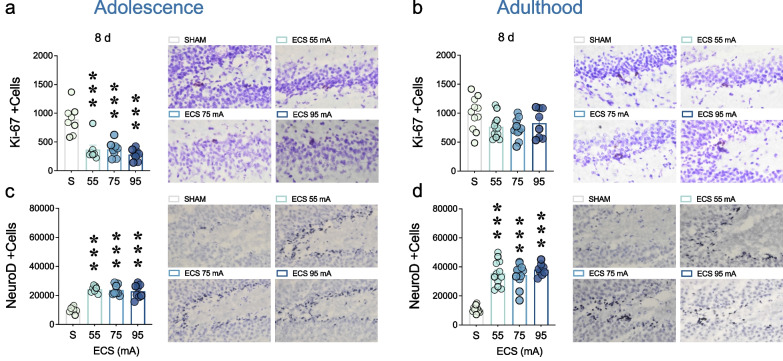
Neurogenic-like effects induced by different intensities of ECS treatment in adolescent and adult female rats. Quantitative analysis of **a**, **b** Ki-67 and **c**, **d** NeuroD + cells in the left dentate gyrus of adolescent or adult female rats. Data represents mean ± SEM of the number of + cells. Individual values are shown for each rat (symbols). The quantification was done in every 8th section through the entire extent of the hippocampal dentate gyrus and multiplied by the sampling factor 8 to provide an estimation of the total number of + cells per marker. One-way ANOVAs followed by Dunnett's multiple comparisons tests: ***p < 0.001 vs. SHAM (S). Representative images for each treatment group showing Ki-67 (brown labeling in the blue granular layer) and NeuroD (dark blue labeling in the blue granular layer) + cells acquired with a light microscope (40× objective lens) connected to a digital camera

ECS exposure (vs. SHAM) decreased Ki-67 + cells as measured 8 days post-treatment in female adolescent rats (F_3,27_ = 18.66, *p* < 0.001; Fig. 4a). In particular, *post-hoc* analysis revealed that ECS decreased hippocampal cell proliferation at all intensities tested (55 mA: − 524 ± 94 Ki-67 + cells, ***p < 0.001; 75 mA: -521 ± 91 Ki-67 + cells, ***p < 0.001; 95 mA: − 612 ± 91 Ki-67 + cells, ***p < 0.001) vs. SHAM-treated adolescent female rats (Fig. 4a). Contrarily, no significant effect was detected for adult female rats (F_3,37_ = 2.75, *p* = 0.056; Fig. 4b).

However, when evaluating the effects of ECS exposure over the survival of neural progenitors (NeuroD + cells), the results showed similar effects both in adolescent (F_3,27_ = 31.53, *p* < 0.001; Fig. 4c) and adult (F_3,38_ = 38.97, *p* < 0.001; Fig. 4d) female rats. For adolescent rats, *post-hoc* analysis revealed that ECS increased the number of hippocampal NeuroD + cells at all intensities tested (55 mA: + 14,233 ± 1758 NeuroD + cells, ***p < 0.001; 75 mA: + 13,584 ± 1698 NeuroD + cells, ***p < 0.001; 95 mA: + 12,809 ± 1698 NeuroD + cells, ***p < 0.001) vs. SHAM-treated adolescent female rats (Fig. 4c). Similarly, but with a higher magnitude of change, *post-hoc* analysis revealed that ECS increased the number of hippocampal NeuroD + cells at all intensities tested (55 mA: + 24,727 ± 2763 NeuroD + cells, ***p < 0.001; 75 mA: + 23,497 ± 2823 NeuroD + cells, ***p < 0.001; 95 mA: + 27,297 ± 3076 NeuroD + cells, ***p < 0.001) vs. SHAM-treated adult female rats (Fig. 4d).

## Discussion

The present study demonstrated that lowering the intensity of the pulse applied during ECS induced an antidepressant-like effect in female adult rats. However, adolescent female rats showed a decreased sensitivity to ECS as compared to adulthood since no beneficial response was observed at any intensity dose tested. These age disparities paralleled some changes detected in the features of the seizures induced by ECS, with adult female rats showing longer tonic and shorter clonic phases, and a much quicker recovery time (almost two-fold) as compared to adolescent female rats. At the neurochemical level some age-particularities were also observed; ECS decreased hippocampal cell proliferation (Ki-67 + cells) in adolescent but not in adult female rats as measured 8 days post-treatment, but for both ages, there was a vast increase in young neuronal survival (NeuroD + cells) by all ECS doses tested. These results reinforce the importance of fine-tuning the parameters of ECS that might render efficacious while considering sex and age as essential variables for treatment response.

During adolescence, ECS did not induce any improvements in female rats as measured in the forced-swim test 1- and 3-days post-treatment. In fact, ECS increased immobility 7 days post-treatment for all doses tested, in line with our previous data [21] and with the negative impact and/or loss of efficacy described for antidepressants in adolescence (e.g., [43, 44]). Interestingly, ECS was capable of inducing an antidepressant-like effect in female adult rats both following 55 or 75 mA, suggesting in line with prior literature, that lower intensities (as opposed to the ineffective dose of 95 mA in females, but effective in males; see [21]) are needed in females to observe a therapeutical response (e.g., [20]). Similarly, to what was observed for adolescent rats, the rates of immobility increased in the ECS groups as measured 7 days post-treatment. In conjunction, these data align with the differences in response to certain antidepressants' efficacy previously reported for males and females [21, 32, 45–47], as well as with prior data reporting that antidepressants differ in efficacy depending on the age of exposure, being adolescence a less responsive period than adulthood (e.g., [21, 35, 43]). The lack of a beneficial effect in adolescence could be attributed to an excessive ECS intensity for such young animals, which might be causing the observed negative effects, in line with previous studies reporting that the electrical charge for effective responses in young females should be lower than the one used for older animals [15–20]. Interestingly, a recent study from our group showed that these young unresponsive female rats, when pretreated with letrozole (an aromatase inhibitor that reduces the biosynthesis of estrogens), displayed improved outcomes for ECS (the 95-mA dose) in terms of inducing an antidepressant-like response in female adolescent rats, suggesting that, besides the dose-intensity used, sex hormones also play a crucial role in the efficacy of the response [48]. Therefore, future studies should evaluate how modulating the dose intensities used relate to how changes in sex hormones affect the antidepressant-like response of ECS. All this information would be key when translating the knowledge acquired into future treatment guidelines to personalize and/or adjust the dose and regimen of ECS to be administered for each sex and age.

In fact, very little evidence is documented in clinical studies that correlated ECT effectiveness and seizure duration; while changes in seizure duration have been measured as a potential marker for ECT treatment efficacy (e.g., [49]), these changes did not appear to be associated with the antidepressant properties of treatment (e.g., [50, 51]). In this context, the present data showed that the 3 intensities tested (55, 75 or 95 mA) did not induce changes in the characteristics of the seizures induced (tonic and clonic phases, recovery time) by the ECS daily dose in adolescent or adult female rats. However, when comparing the results by age, adult female rats showed different characteristics of the seizures induced as compared to adolescent rats: slightly longer tonic phase, paired with a shorter clonic phase, and a faster recovery time (almost two-fold quicker) in adulthood. Interestingly, the lowest dose-intensities tested (55 and 75 mA), that also induced an antidepressant-like effect in adult female rats, showed significantly lower clonic times than adolescent rats, suggesting a possible role for the type of seizure induced in the age-related behavioral responses. Thus, future experiments will center in evaluating alternative dosing parameters for the electrical charge and seizure threshold that could potentially render effective in female adolescent rats.

Given the age-specific differences in the antidepressant-like responses induced by different dose-intensities of ECS in female rats, we then evaluated the early stages of hippocampal neurogenesis (i.e., cell proliferation and neural progenitors) as a possible mechanism behind these age-disparities. In particular, the rate of cell proliferation was decreased in female adolescent rats treated with all dose-intensities of ECS, and as measured 8 days post-treatment, but not in adult rats. These results align with our previous findings showing that repeated ECS (dose intensity of 95 mA) induced an early increase in cell proliferation (observed 1 day post-treatment) that later led to a decrease in the number of Ki-67 + cells, as observed 8 days post-treatment in adolescent male or female rats, or 15 days post-treatment in adult male rats (see [21]), while describing similar magnitude results (not dose-related) in female rats treated with other ECS intensities (55 and 75 mA). Therefore, the decrease observed post-treatment could be the result of an adaptive time-course mechanism following an initial excessive increase, and/or, as previously discussed (see [21]), the consequence of a series of stressful forced-swim tests leading to a drop in cell proliferation, since stress impairs hippocampal neurogenesis. Moreover, ECS (also independently of the intensity applied) increased hippocampal neural progenitors as labeled by NeuroD + cells, and measured 8 days post-treatment, in female adolescent and adult rats. As discussed in more detail in our prior publication (see [21]), although this excitatory pro-neurogenic activity is traditionally considered beneficial, one could not exclude the possibility that seizures might generate misplaced neurons with irregular morphological and electrophysiological features, such as the ones observed in epileptic rodent models, a process described as aberrant neurogenesis (e.g., [52, 53]), and whose functions stills need to be defined. In fact, since newly generated proliferating cell and neural progenitors were only partially needed for ECS' antidepressant-like response to occur (see [21]), the changes induced by ECS might contribute to other roles in the hippocampus (see [54]), such as promoting structural plasticity (e.g., reviewed by [55]), gliogenesis [56], synaptogenesis (e.g., [57]) and angiogenesis [58], that might even be mediating some of the long-term consequence induced by ECS (e.g., [14]), and that deserve further characterization. Overall, the present data, together with our prior study [21], report that different dose-intensities of ECS modulated hippocampal neurogenesis in a comparable fashion with age (i.e., decreased cell proliferation observed 8 days post-treatment for adolescent rats, and expected 15 days post-treatment for adults; increased survival of neural progenitors 8 days post-treatment). Therefore, since ECS only rendered efficacious in adult female rats, but neurogenesis was regulated similarly for both ages, the observed antidepressant-like response in adulthood might be in part driven by other molecular mechanisms (e.g., monoaminergic transmission, e.g., [59]; neurotrophic changes, e.g., [60, 61]) in hippocampus or other brain regions (e.g., see recent reviews by [62, 63]).

## Perspectives and significance

These results proved that decreasing the intensity of the pulse applied during ECS rendered effective by inducing an antidepressant-like effect in adult female rats, but was insufficient in adolescence. The lack of efficacy observed in adolescence might be explained by differences in the characteristics of the seizures induced by ECS as compared to adulthood. Moreover, the early neurogenic-like capabilities induced by ECS, observed both in adolescence and adulthood, go beyond the regulation of its antidepressant-like effects and deserves a broaden characterization. The present data reinforce the need of fine-tuning the parameters of ECS to render efficacy when considering sex and age as essential variables for treatment response.

## Data Availability

The datasets used and/or analyzed during the current study can be made available from the corresponding author on reasonable request.
